# Regulation of cortical hyperexcitability in amyotrophic lateral sclerosis: focusing on glial mechanisms

**DOI:** 10.1186/s13024-023-00665-w

**Published:** 2023-10-19

**Authors:** Manling Xie, Praveen N. Pallegar, Sebastian Parusel, Aivi T. Nguyen, Long-Jun Wu

**Affiliations:** 1 Department of Neurology, Mayo Clinic, 200 First Street SW, Rochester, MN 55905 USA; 2 Mayo Clinic Graduate School of Biomedical Sciences, Rochester, MN USA; 3 Department of Laboratory Medicine and Pathology, Mayo Clinic, Rochester, MN USA; 4 Department of Neuroscience, Mayo Clinic, Jacksonville, FL USA; 5 Department of Immunology, Mayo Clinic, Rochester, MN USA

## Abstract

Amyotrophic lateral sclerosis (ALS) is a progressive neurodegenerative disorder characterized by the loss of both upper and lower motor neurons, resulting in muscle weakness, atrophy, paralysis, and eventually death. Motor cortical hyperexcitability is a common phenomenon observed at the presymptomatic stage of ALS. Both cell-autonomous (the intrinsic properties of motor neurons) and non-cell-autonomous mechanisms (cells other than motor neurons) are believed to contribute to cortical hyperexcitability. Decoding the pathological relevance of these dynamic changes in motor neurons and glial cells has remained a major challenge. This review summarizes the evidence of cortical hyperexcitability from both clinical and preclinical research, as well as the underlying mechanisms. We discuss the potential role of glial cells, particularly microglia, in regulating abnormal neuronal activity during the disease progression. Identifying early changes such as neuronal hyperexcitability in the motor system may provide new insights for earlier diagnosis of ALS and reveal novel targets to halt the disease progression.

## Introduction

Since Charcot originally described the neuroanatomical pathology of amyotrophic lateral sclerosis (ALS) in 1874 [1], growing clinical and scientific interest has emerged in the ALS field. ALS is a rare disease, with an estimated prevalence of 5.5 cases per 100,000 people in the US, 2.6–3.0 in Europe, and 0.8–1 in Asia [2–8]. The age of ALS onset is 51–66 years, with a later onset in European patients (65 years) [2, 9]. Sex is another risk factor for ALS, with a male-to-female ratio between 1:1 and 2:1 [4, 10–13]. Over 90% of ALS cases are sporadic (sALS), meaning they occur without a family history. Less than 10% of ALS cases are familial (fALS) and can be traced to inherited ALS-causing mutations [14]. More than 120 ALS-related genetic variants have been identified [15], including genetic mutations in *C9orf72* (~ 40%, encoding chromosome 9 open reading frame 72), *SOD1* (~ 12%, encoding superoxide dismutase 1), *TARDBP* (~ 5%; encoding TAR DNA-binding protein 43, TDP43), and *FUS* (~ 4%; encoding RNA binding protein fused in sarcoma) being the most common [16]. Diagnosing ALS can be challenging due to its varied presentation and similarities with other neurological conditions. The current ALS diagnosis includes comprehensive assessment of clinical symptoms, electromyography (EMG) studies, nerve conduction study, muscle and nerve biopsy and exclusion of other conditions that can mimic ALS [2, 17]. Neuroimaging techniques, such as magnetic resonance imaging (MRI) and positron emission tomography (PET), have shown promise in aiding ALS diagnosis. As of now, there is no cure for ALS, and treatment primarily focuses on managing symptoms, slowing disease progression, and improving the quality of life for patients. Currently, there are two FDA-approved medications for the treatment of ALS. They are Riluzole, functioning by reducing the release of glutamate, and Edaravone, designed to alleviate oxidative stress. Despite their availability, both drugs yield only modest advantages in terms of extending survival and delaying disease progression. Consequently, advancing our understanding of ALS pathogenesis and developing more effective, personalized treatments are essential to improve the lives of individuals living with this devastating condition.

**Fig. 1:**
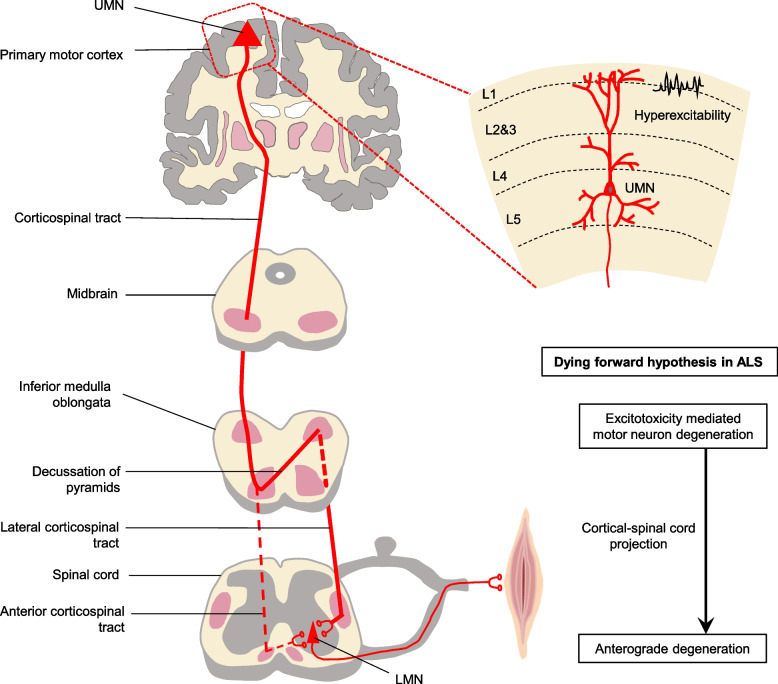
The dying forward hypothesis in ALS. The dying-forward hypothesis suggests that the dysfunction and death of motor neurons, (especially involving Betz cells), begins in the motor and pre-motor cortices and then progresses downwards to lower motor neuron (LMN) through an anterograde pattern along the cortical-spinal cord projection, leading to motor neuronal death in the brainstem and spinal cord. Excitotoxicity due to the hyperexcitability is an important mediator of upper motor neuron (UMN) dysfunction, but not the only factor

Although Charcot described corticospinal tract degeneration and motor neuron loss as two neuropathological hallmarks in ALS, the origin of ALS is still debated. The “dying-forward hypothesis” proposes that neurodegeneration initiates in cortical motor and pre-motor areas (especially involving Betz cells) and then progresses to lower motor areas through an anterograde pattern, causing neuronal death in the brainstem and spinal cord [18–20] (Fig. 1). The critical evidence supporting this hypothesis is the clinical observation of cortical hyperexcitability as an early feature in ALS patients, preceding the cascade of other hallmark pathologies, such as the formation of abnormal protein aggregates, robust glial cell activation, and eventually motor neuron death [21–25]. Cortical hyperexcitability has also been confirmed in preclinical animal and cell models [26–28], indicating this phenomenon is a common feature and can serve as a biomarker for early detection.

The mechanisms underlying motor neuron hyperexcitability are not fully understood. Both cell-autonomous and non-cell-autonomous mechanisms are believed to be involved. The cell-autonomous mechanisms refer to the change of motor neuron intrinsic electrophysiological properties, leading to their enhanced excitability. Upper motor neurons (UMNs), or cortical motor neurons, are larger pyramidal cells located within the fifth layer in the primary motor cortex, and are anatomically and functionally different from other neurons. Due to their unique molecular basis of cell-intrinsic pathways, they exhibit an increased likelihood to fire action potentials in response to certain stimuli and display selective vulnerability in the context of ALS [29–31]. In the motor cortical circuits, UMNs receive signals from cortical intratelencephalic neurons, local inhibitory neurons, as well as from long projecting neurons from distal brain regions [32, 33]. As a result, their activity is shaped by neuronal network in the cortex. Non-cell autonomous mechanisms mediated by glial cells have also been increasingly recognized recently. Glial cells, including astrocytes, oligodendrocytes and microglia, display a complex repertoire of functional changes during disease progression. However, the exact mechanisms how they contribute to neuronal hyperexcitability in the context of ALS are not fully understood. To review the current progress on this topic (particularly glial mechanisms), we searched through the PubMed, EMBASE, and Web of Science electronic databases to identify relevant recent studies, using a combination of MeSH terms and keywords: “Amyotrophic Lateral Sclerosis”, “Cortical excitability”, “Glia cell”, “Microglia”, “Astrocyte” and “Oligodendrocyte”. We summarize the current clinical and preclinical evidence of cortical hyperexcitability and the underlying mechanisms, highlighting glial regulation of neuronal network excitability in ALS.

## Cortical hyperexcitability is a common feature of ALS

Hyperexcitability refers to the change of neuron excitability, rendering the neurons more prone to fire action potentials in response to stimuli. Motor cortical hyperexcitability is a common phenomenon reported in both ALS patients [22–25, 34] and ALS rodent models [26–28]. The two current FDA-approved drugs for ALS, Riluzole and Edaravone [35, 36], preferentially block sodium channels [37, 38] and glutamate receptors respectively [39]. These two drugs can inhibit neuronal excitability, suggesting a detrimental role of motor neuron excitotoxicity in ALS.

### Cortical hyperexcitability in ALS patients

A plethora of clinical studies have revealed that cortical hyperexcitability occurs at the symptomatic stage in both sALS and fALS patients [40, 41]. Cortical excitability can be clinically assessed by different techniques, including threshold tracking paired-pulse transcranial magnetic stimulation (TMS), electroencephalography (EEG), magnetoencephalography (MEG) and functional MRI (fMRI) [42–44].

TMS is the most widely used technique to assess cortical excitability in ALS. It is a noninvasive form of cortical stimulation that applies a changing magnetic field outside the brain to affect central nervous system (CNS) activity [45, 46]. Based on the frequency, duration, and intensity (amplitude) of stimulation and the coil type, different TMS parameters indicative of cortical excitability can be recorded. The definition and indication of TMS parameters and how these parameters change in ALS patients have been summarized in Table 1 [45, 47–51]. Overall, TMS measurements indicate that motor cortical hyperexcitability is a common feature of ALS preceding the symptomatic stage, characterized by reduced motor evoked potentials and motor threshold, increased central motor conduction time, shortened cortical silent period, reduced short-interval intracortical inhibition, and increased intracortical facilitation [45]. In addition, this evidence indicates that both excitatory and inhibitory neuronal dysfunction are involved in motor cortical hyperexcitability. TMS can be combined with neuroimaging studies (fMRI) to detect the cortical hyperexcitability. fMRI measures changes in blood oxygenation and flow in the brain in response to specific tasks or stimuli, which reflects the neural activity and connectivity in the respective region. Resting state fMRI indicates enhanced network connectivity of the motor cortex with multiple brain regions in ALS [52, 53], which was also confirmed by EEG and MEG recordings [54–56].

**Table 1:** Summary of TMS parameters indicative of cortical excitability change in ALS patients

TMS parameters	Definition	Indicative	Change in ALS	Reference
Motor evoked potentials (MEP)	Electrical signals recorded from descending motor pathways or from muscles after stimulation of motor pathways within the brain	Integrity of the descending corticospinal tract	Reduced	[45]
Motor Threshold (MT)	Minimal intensity of motor cortex stimulation required to elicit a reliable MEP of minimal amplitude in the target muscle	Cortical motor neuronal membrane excitability	Reduced (early) Increased (late)	[47]
Central motor conduction time (CMCT)	Time required for neural impulses to travel through the central nervous system on their way to the target muscles	Conduction between the primary motor cortex and spinal cord	Increased	[48]
Cortical silent period (CSP)	Temporary interruption of electromyographic signal from a muscle following a MEP triggered by TMS over the primary motor cortex (M1)	M1 function. The CSP is typically shortened if M1 is affected, but often grossly prolonged if areas outside M1	Normal or shortened	[49]
Short interval intracortical inhibition (SICI)	Inhibition of MEPs upon a subthreshold conditioning stimulus applied at predetermined short time intervals (7-10 ms) prior to a test stimulus	Balance between inhibition and facilitation. Reduction or absence of SICI is a biomarker of cortical inhibitory GABAergic neuron dysfunction	Reduced	[50]
Intracortical facilitation (ICF)	Increased excitability can be elicited with a similar protocol as SICI but at longer interstimulus interval of 6–30 ms	Intracortical synaptic excitability	Increased	[51]

Immunohistochemistry and whole-genome sequencing using postmortem ALS patient tissues also provided evidence of cortical hyperexcitability, including the dysregulation of NMDA receptor and AMPA receptors in primary motor cortex [57], as well as increased glutamate levels [58–60]. Loss of inhibitory neurons has also been reported in ALS [61], in addition to the downregulation of GABA receptors [62] and GABA levels [63, 64]. Taken together, clinical evidence from electrophysiology, neuroimaging, and gene sequencing has revealed cortical hyperexcitability in ALS patients at the pre-symptomatic stage. However, the clinical measurements are insufficient to decode the mechanisms behind the cortical hyperexcitability at the cellular and molecular level.

### Cortical hyperexcitability in ALS related mouse models

Transgenic mouse models are valuable tools to monitor the neuronal excitability at different disease stages and identify the underlying pathological mechanisms. A variety of ALS mouse models were generated by expressing ALS-related mutations, most commonly in *SOD1* and *TDP-43* [65–68]. Multiple techniques can be used to study the excitability of motor neurons in mouse models, including electrophysiological techniques (such as patch clamp recording) and neuroimaging techniques (such as two-photon in vivo imaging). RNA-sequencing can provide information regarding gene expression patterns of the specific cell types that are involved in regulating motor neuron excitability, providing insights into the mechanisms of neuronal hyperexcitability in ALS.

Motor neuron hyperexcitability has been observed in different ALS transgenic mouse models by patch-clamp recordings. For example, recordings of membrane potential in the *SOD1*^*G93A*^ mouse model showed increased motor neuron excitability, characterized by increased firing frequency and shorter duration [69]. Using the same model, a later study further confirmed increased synaptic excitation in motor cortex layer V pyramidal neurons at the presymptomatic stage [26, 27]. A similar phenomenon has also been reported in the TDP-43^Q331K^ mouse model [70]. In addition, hyperexcitability has also been confirmed by recording *SOD1*^*A4V/*+^ ALS patient-derived motor neurons [23].

Two-photon in vivo calcium imaging is a powerful tool to study cortical hyperexcitability in awake animals. Specifically, different types of neurons can be labeled by the expression of calcium indicators, which have a high detection rate for single action potentials [71]. In awake *hSOD1*^*G93A*^ mice, motor neuron activity was monitored by expressing calcium indicator GCaMP6s [27]. Although there were no significant differences in the overall frequency of activity, amplitude, or the time course of individual events at P90-P129, basal calcium level was elevated in *hSOD1*^*G93A*^ mice, indicating impaired calcium homeostasis [27]. Future studies using two-photon in vivo imaging to perform longitudinal studies monitoring the neuronal activity during the disease progression are needed. This will allow to focus on different types of cells in different brain areas, which will provide better insights into the underlying mechanism of neuronal hyperexcitability in ALS.

RNA-seq has been widely used to analyze gene expression patterns in the cortex of ALS patients and ALS mouse models. The results provide evidence of changes in gene expression patterns that are associated with cortical hyperexcitability. For example, changes in expression of ion channels and specific glutamate receptor subunits have been observed in the *hSOD1*^*G93A*^ mouse model [27]. These changes suggest that disruptions in ion channel function may underlie the intrinsic electrophysiological properties of motor neurons in ALS.

## The potential mechanisms underlying motor cortical hyperexcitability in ALS

**Fig. 2:**
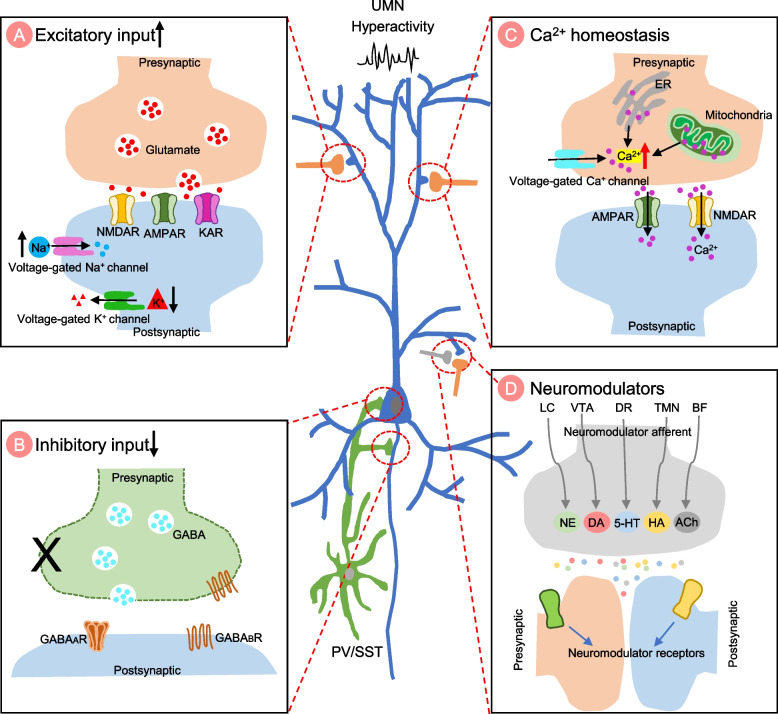
The cell-autonomous mechanisms of UMN hyperexcitability in ALS. Cell-autonomous mechanisms refer to the change in intrinsic properties of upper motor neurons (UMNs) that contribute to the hyperexcitability. **A** Representation of an excitatory synapse in ALS. Glutamatergic signaling mediated UMN hyperexcitability can be caused by various mechanisms: 1) increased expression levels and/or modifications of the glutamate receptors [such as N-methyl-D-aspartate receptor (NMDAR), α-amino-3-hydroxy-5-methyl-4-isoxazolepropionic acid receptor (AMPAR) and kainate receptor (KAR)]; 2) increased presynaptic glutamate release and, 3) decreased glutamate removal by glial cells. Ion channel dysfunctions also contribute to hyperexcitability, such as increased inward Na^+^ current and decreased outward K^+^ current, resulting in an increased repetitive firing of UMNs. **B** Representation of an inhibitory synapse in ALS. Loss or dysfunction of inhibitory neurons and alteration of inhibitory neurotransmitter receptors (such as GABAR) impaired inhibitory circuits, leading to UMN hyperexcitability. **C** Representation of impaired Ca^2+^ homeostasis in synapse in ALS. Increased cytosolic Ca ^2+^ triggers presynaptic glutamate release and regulates postsynaptic glutamate receptors efficiency, contributing to UMN hyperexcitability. The mechanisms underlying abnormal Ca^2+^ homeostasis include abnormal expression of Ca^2+^ permeable channels and pumps, dysfunction of endoplasmic reticulum (ER) and mitochondrial due to oxidative stress, and an impaired Ca^2+^ buffering system. **D** Representation of neuromodulator regulation in synapses in ALS. The neuromodulator regulation is crucial for fine-tuning and coordinating complex motor cortical circuits. Changes in neuromodulator levels may also be involved in the development of cortical hyperexcitability in ALS

**Fig. 3:**
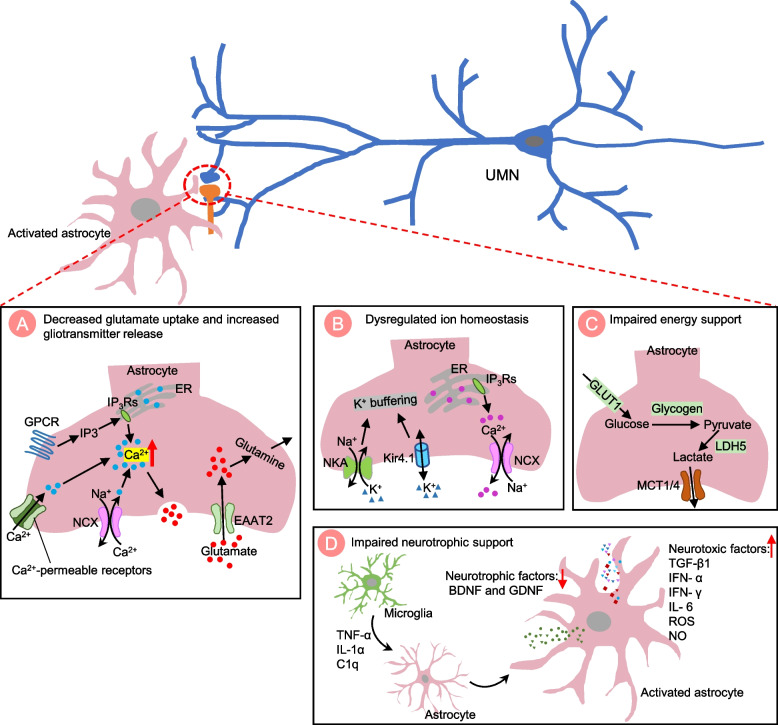
The non-cell-autonomous mechanisms of UMN hyperexcitability in ALS: astrocyte dysfunction. Representation of astrocyte-neuron interaction in ALS. **A** Defective astrocytes lead to an accumulation of glutamate in the synaptic cleft, resulting from both a loss of glutamate uptake through astrocyte excitatory amino acid transporters (EAAT1/GLAST and EAAT2/GLT1) and an excessive release of glutamate caused by disruption of Ca^2+^ homeostasis in astrocytes. These defects contribute to hyperexcitability of upper motor neurons (UMNs). Impaired Ca^2+^ homeostasis in astrocytes may be due to increased permeability of Ca^2+^ receptors/channels (transient receptor potential ankyrin 1 (TRPA1), AMPA, NMDA and P2X receptors), increased Ca^2+^ release from internal stores (endoplasmic reticulum, ER) via activation of G protein-coupled receptors (GPCRs) and reversed operation of the sodium-calcium exchanger NCX. **B** Defective astrocytes cause an ionic imbalance in the synaptic cleft due to dysfunction of astrocytic Kir4.1 channels and sodium–potassium-ATPase (NKA). This results in an accumulation of K^+^ in the synaptic cleft, which leads to abnormal firing of neurons. Activity-dependent Na^+^ transients switch NCX to a “reverse mode” by mediating Ca^2+^ import and Na^+^ export. **C** Defective astrocytes become less efficiency in providing metabolic support for neurons. Disrupting the expression of the astrocytic lactate transporters, monocarboxylate transporter 4 (MCT4) or MCT1, results in decreased lactate support for neurons. **D** Defective astrocytes release lower levels of neurotrophic factors [such as brain-derived neurotrophic factor (BDNF) and glial cell line-derived neurotrophic factor (GDNF)] and produce abnormal levels of cytokines and other toxic substances [such as transforming growth factor beta 1 (TGF-β1), interferon alpha (IFN- α), interferon gamma (IFN- γ), interleukin 6 (IL- 6), reactive oxygen species (ROS), and nitric oxide (NO)]. Microglia produce tumor necrosis factor alpha (TNF-α), interleukin 1 alpha (IL-1α), and complement component 1q (C1q) to trigger the activation of neurotoxic reactive astrocytes

**Fig. 4:**
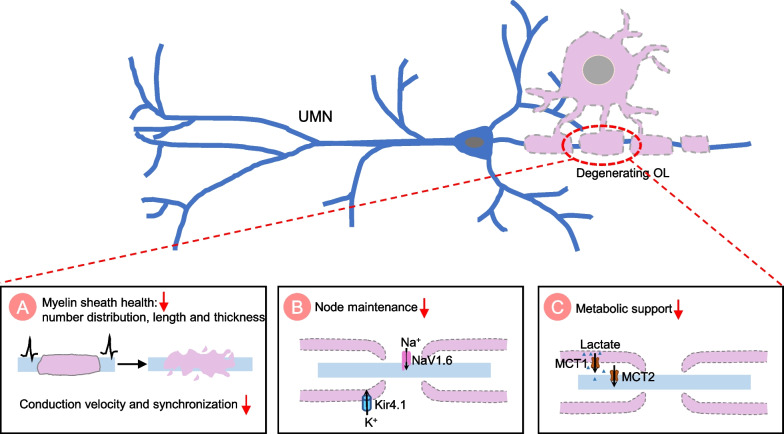
The non-cell-autonomous mechanisms of UMN hyperexcitability in ALS: Oligodendrocyte dysfunction. Representation of oligodendrocyte-neuron interaction in ALS. **A** Defective oligodendrocytes can lead to myelin degeneration, resulting in changes in the number, distribution, length, and thickness of myelin sheaths. This directly disrupts action potential conduction along the axon and alters upper motor neuron (UMN) excitability. **B** Defective oligodendrocytes become less effective in regulating the composition and structure of the nodes of Ranvier. Demyelination induced paranodal junction destabilization leads to a significant disruption of Na^+^ channel clustering in the node. Oligodendrocytes also lost their ability to regulate white matter K^+^ buffering by clearing extracellular K^+^ through Kir4.1 channels. **C** Defective oligodendrocytes become less effective in providing metabolic support to axons through lactate transporters

**Fig. 5:**
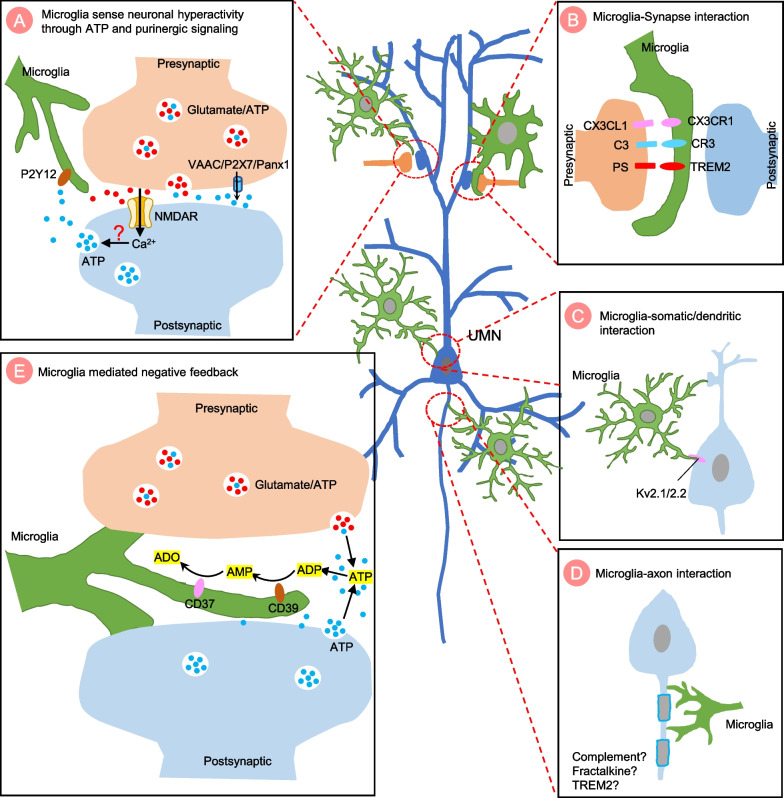
The potential function of microglia in sensing and regulating UMN hyperexcitability in ALS. Representation of microglia-neuron interaction in ALS. **A** Microglia may sense upper motor neuron (UMN) hyperexcitability through ATP and purinergic signaling. Hyperactivated motor neurons release ATP into the extracellular space through channel-mediated release mechanisms [such as volume-activated anion channels (VAAC), P2X7 receptors, and Pannexin 1 channels (Panx1)] or through Ca.^2+^-dependent vesicular release mechanism. Extracellular ATP is then hydrolyzed into ADP and sensed by P2Y12 receptors, recruiting microglia processes towards this ATP source. **B** Activated microglia interact with dendritic spines and trigger synaptic pruning. The molecular components involved in synaptic pruning may include complement proteins (such as C3/CR3), CX3CL1/CX3CR1 and TREM2/lipid phosphatidylserine (PS). **C** Activated microglia contact neuronal dendrites or somatic membranes at Kv2.1/2.2 clustering through microglial P2Y12 receptor to for purinergic junctions. **D** Microglia interact with the axon initial segment to regulate action potential generation through mechanisms that are currently unknown. **E** Extracellular ATP can be converted to AMP by the microglial ATP/ADP-hydrolyzing ectoenzyme CD39. AMP can then be used as a substrate for the microglial enzyme CD73 to generate adenosine (ADO), which can suppress neuronal activity by binding to adenosine A1 receptors (A1Rs)

The mechanisms underlying cortical hyperexcitability are not fully understood, but it is believed to involve a complex interplay between cell-autonomous and non-cell-autonomous factors. Change of motor neuron intrinsic electrophysiological properties can trigger hyperexcitability through different mechanisms, including the imbalance of excitatory/inhibitory input, impairment of Ca^2+^ homeostasis, and alteration of neuromodulator regulation (Fig. 2). Glial cell mediated non-cell-autonomous mechanisms also contribute to UMN hyperexcitability (Figs. 3, 4 and 5). The precise mechanisms may vary depending on disease stages and types of ALS.

### Cell-autonomous mechanisms

#### Increased excitatory input

Increased excitatory input to UMNs could be due to both intrinsic and extrinsic alterations (Fig. 2A). Intrinsically, the electrophysiological properties of UMNs are altered in ALS, including excitatory neurotransmitter receptors and ion channels involved in action potential generation. For example, the glutamate receptors are reported to be upregulated in ALS. In the frontal cortex in ALS cases, neurotransmission- and synaptic-related genes are extensively up-regulated, including genes coding for AMPA receptor (*GRIA1*), and NMDA receptors (*GRIN2A* and *GRIN2B*) [72]. *GRIA1* upregulation has been confirmed by another clinical study, whereas *GRIA2* and *GRIA3* were reduced in a subset of ALS patients [57]. In addition, alterations of post-transcriptional modification of glutamate receptors also affects UMN excitability. For example, presymptomatic *SOD1*^*G93A*^ mice exhibit a selective decrease of NMDAR subunits GluN2A expression and autophosphorylation at threonine-286 in upper motor neurons synapses, indicating an alteration in the synaptic plasticity [73]**.**

In addition to the alterations in glutamate receptors, ion channel abnormalities have also been implicated in triggering neuronal hyperexcitability. For example, the persistent sodium (Na^+^) current was significantly higher in the *SOD1* mouse model [74]. A similar phenomenon has been confirmed in both sALS [22] and fALS patients [75], which could directly increase motor neuron excitability. Interestingly, in ALS patients, the K^+^ conductance is reduced, further contributing to motor neuron hyperexcitability. Investigations of axonal excitability properties have confirmed that the persistent Na^+^ conductance is increased in conjunction with a decline in K^+^ conductance in ALS patients [76]. Consistently, reduced expression of axonal potassium channels was observed by immunoreactivity of potassium channels (Kv1.2) in ALS patients [77] and motor neuron gene expression analysis [78]. Altogether, these studies suggest upregulated persistent Na^+^ conductance and decreased K^+^ conductance in ALS patients and that these alterations are intrinsically associated with motor neuron hyperexcitability.

Extrinsically, an increase in presynaptic release and/or decreased removal, results in an excess glutamate in the synaptic cleft of UMNs in ALS. UMNs receive excitatory synaptic input primarily from cortical intratelencephalic neurons [79]. Motor cortical intratelencephalic neurons exhibit increased activity in ALS [27], which project excess excitatory input to motor neurons, leading to hyperactivity of motor neurons. To a lesser degree, UMNs also receive excitatory input from Layer V intratelencephalic and long-range input from other brain regions, including the contralateral primary motor cortex, secondary motor cortex, frontal and somatosensory cortex and auditory cortex, as well as the thalamus [80]. Whether UMNs receive more excitatory inputs from these brain areas needs further investigation.

In addition to increased release of excitatory neurotransmitters, the removal of glutamate by glial cells is impaired in ALS [81–85]. Glutamate-mediated excitotoxicity is prevented by rapid clearance of excess glutamate located in the synaptic cleft through glutamate transporters, mainly expressed by astrocytes and neurons. The activity and expression levels of glutamate transporters are reduced in ALS, leading to glutamate accumulating in the synaptic cleft [60]. Thus, increased excitatory input via receptor upregulation and the excess glutamate in the extracellular space likely leads to hyperexcitability of motor neurons.

#### Decreased inhibitory input

Inhibitory input is mainly mediated by the inhibitory neurotransmitter GABA (gamma-aminobutyric acid). UMNs receive inhibitory input from parvalbumin (PV) and somatostatin (SST) interneurons. Clinical observations from TMS reveal a reduction or a complete loss of short interval intracortical inhibition, which indicates dysfunction of interneurons in ALS. There are several factors that can disrupt the inhibitory circuits, including loss of inhibitory neurons, compromised inhibitory neuron function, and alteration of GABA receptors (Fig. 2B). The current findings regarding the density of interneurons in the motor cortex are inconsistent. For example, a previous study found that cortical interneurons, and other projecting neuron populations, are not affected in the motor cortex in *hSOD1*^*G93A*^ mice at the presymptomatic stage [86]. However, using the same mice, another group found that neuropeptide Y-expressing (NPY) interneurons are significantly decreased at symptom onset, while the number of calretinin-expressing (CR) interneurons is progressively reduced during later symptomatic stages to end-stage [87]. In addition, a reduction of parvalbumin- and somatostatin-positive inhibitory interneurons has also been reported in the wobbler mouse model of ALS [88]. More recently, pronounced reductions of inhibitory synapses have been reported in the *Fus*^ΔNLS/+^ mouse model by ultrastructural analysis, indicating an inhibitory synaptic defect [89].

Impaired inhibitory neuron function has also been reported in various ALS mouse models. For example, L5-PV + interneurons were hypoactive in the *SOD1* mouse model during the late presymptomatic stage of the disease (8.5–10 weeks) [61]. However, another group reported that the intrinsic excitability of PV interneurons was increased in the *hSOD1*^*G93A*^ mice at P90–P101 [27]. In addition, increased activity of layer V interneurons in the motor cortex was able to reduce UMN hyperexcitability, delay the onset of motor deficits, and slow motor neuron degeneration and symptom progression in *hSOD1*^*G93A*^ mouse model [61], which further confirms the critical role of the inhibitory circuitry. The decreased inhibitory neuron density and impaired inhibitory neuron function can directly lead to a reduction in GABA levels. Indeed, the reduced GABA concentration in the brain has been reported in ALS patients by using proton magnetic resonance spectroscopy (1H-MRS) [63]. These observations collectively indicate that the inhibitory neuron function exhibits variable changes throughout the course of the disease.

Regulations in the expression and modification of postsynaptic GABAergic receptors have also been reported in ALS. For instance, gene expression of GABA receptors *GABRA1, GABRD, GABRB2* was significantly increased in ALS patients, which may reflect a compensatory mechanism [72]. However, downregulation of six subunits of the GABA_A_ receptor has also been reported in a subset of ALS patients [57]. This seemingly contradictory observation might be due to the different types of ALS.

Altogether, motor neurons shift towards a more excitatory state due to increased excitatory input and decreased inhibitory input. Further research is needed to fully understand the mechanisms underlying the alterations in the balance of excitation and inhibition in ALS and to determine the extent to which this contributes to the motor neuron hyperexcitability. This information may provide new insights into the development of new therapeutic strategies aiming to restore the balance between excitatory and inhibitory signaling.

#### Ion homeostasis impairment: Ca^2+^ and K^+^

In the CNS, Ca^2+^ homeostasis is essential for neuronal excitability. Motor neurons have a low Ca^2+^ buffering capacity, and the cytoplasmic calcium concentration is tightly regulated [90]. In the resting condition, the cytoplasmic Ca^2+^ concentration is maintained at around 100 nM, which is above 10,000-fold lower than the extracellular concentration. In the intracellular space, the endoplasmic reticulum (ER) and mitochondria act as the main Ca^2+^ sinks. Abnormalities in calcium homeostasis have been widely implicated in the pathogenesis of ALS [91, 92]. The potential mechanisms underlying the impairment of Ca^2+^ homeostasis include abnormal Ca^2+^ permeable channels and pumps, ER and mitochondrial dysfunction, and an impaired Ca^2+^ buffering system (Fig. 2C) [91].

Firstly, the expression level and function of Ca^2+^ channels and pumps, which control the influx and efflux of Ca^2+^, are reported to be altered in ALS. For example, defective GluA2 (glutamate AMPA receptor subunit 2) mRNA editing increases AMPA receptor Ca^2+^ permeability, further changing the motor neuron excitability in ALS patients [93–95]. Secondly, ALS is often associated with mitochondrial [96–99] and ER dysfunction [100–103] due to the increased oxidative stress. As a result, mitochondria and ER lose their Ca^2+^ buffering ability. Thirdly, reduction of Ca^2+^ binding proteins parvalbumin and calbindin D28k at early stage in large motor neurons has been reported in ALS patients [104]. This was further confirmed in *SOD1* transgenic mice [105], indicating an impairment of Ca^2+^ buffering system. Thus, Ca^2+^ dysregulation may contribute to motor neuron hyperexcitability in ALS.

The homeostasis of extracellular K^+^ concentration also affects motor neuron excitability, especially as the excess K^+^ in the extracellular space can directly lead to synchronized neuronal activity [106]. The extracellular K^+^ homeostasis is mainly maintained by astrocytes through potassium uptake and spatial buffering. In ALS, astrocytes lose their K^+^ buffering ability which is discussed in the next section.

#### Neuromodulators

Neuromodulators are chemicals that are not directly involved in generating action potentials by activating ionotropic receptors. They act together with neurotransmitters to regulate excitatory and inhibitory synaptic function by altering the strength of synaptic transmission (Fig. 2D). The five major small molecular neuromodulators include dopamine, serotonin, noradrenaline, histamine, and acetylcholine. Motor neurons receive neuromodulators from specific neuronal populations located in distinct brain regions, including the noradrenergic neurons in the locus coeruleus (releasing norepinephrine NE), dopaminergic neurons in the ventral tegmental area (releasing dopamine DA), serotonergic neurons in the dorsal raphe nucleus (releasing serotonin 5-HT), cholinergic neurons in the basal forebrain (releasing acetylcholine ACh), and histaminergic neurons of the tuberomammillary nucleus in the hypothalamus (releasing histamine HA) [107]. This neuromodulator regulation is crucial for the fine-tuning and coordination of complex motor cortical circuits. ALS patients show alterations in these neuromodulator pathways (comprehensively reviewed in [107]). As a result, changes in levels of neuromodulators may also be involved in the development of cortical hyperexcitability in ALS.

### Glial cells mediate non-cell autonomous mechanisms in UMN hyperexcitability

Glial cells play critical roles in maintaining proper neuronal activity by regulating neurotransmitter levels, modulating synaptic transmission/plasticity, and providing metabolic support and so forth. However, our understanding of how glial cells respond to and regulate neuronal activity in brain diseases, particularly neurodegenerative disease, is very limited. In this section, we summarize the current evidence regarding glia-neuron interaction in ALS, and their potential role in regulation of UMN hyperexcitability.

#### Astrocytes

Astrocytes perform many supportive functions that are important for maintaining the proper functioning of the nervous system, including metabolic support, blood–brain barrier maintenance, and waste product clearance [108, 109]. Importantly, astrocytes play a key role in regulating neuronal activity by closely associating with neuronal synapses, regulating neurotransmitter uptake and release, maintaining the extracellular ion balance, and modulating synaptic transmission [108, 109]. In ALS, astrocytes undergo robust activation, characterized by changes in both their morphology and function [110]. A growing body of evidence suggests that astrocyte dysfunction may contribute to the development and progression of ALS, eventually causing motor neuron degeneration [110–116]. The function and therapeutic potential of astrocytes in ALS has been comprehensively reviewed previously [110]. Here, we mainly discuss how dysfunctional astrocytes with a neurotoxic phenotype contribute to motor neuron hyperexcitability (Fig. 3).

Firstly, defective astrocytes result in excessive accumulation of glutamate in the synaptic cleft (Fig. 3A). In physiological conditions, astrocytes can prevent excitotoxicity by uptaking excess glutamate in the synaptic cleft efficiently through sodium-dependent excitatory amino acid transporters (EAAT1/GLAST) and EAAT2 (GLT1 in mice). EAAT2/GLT1 is exclusively expressed in astrocytes and response for 90% glutamate uptake [85]. Defects in glutamate uptake by loss of astrocyte EAAT2/GLT1 has been found in both ALS patients and related mouse models [81–84]. In addition, astrocytes also release gliotransmitter including glutamate through a Ca^2+^ dependent manner [117]. Excess glutamate release evoked by interruption of Ca^2+^ homeostasis in astrocytes has been reported in ALS [118–121]. Altogether, excessive accumulation of glutamate in the synaptic cleft, may trigger excitotoxicity and neurodegeneration in ALS.

Secondly, astrocyte dysfunction disrupts ion homeostasis in the synaptic cleft (Fig. 3B). Astrocytes help to clear excess K^+^ and Ca^2+^ from the synaptic cleft, preventing their accumulation and maintaining proper synaptic transmission. Reduction of the K^+^-buffering ability of astrocytes has been reported in ALS [106, 122, 123]. Progressive loss of astrocyte potassium channel Kir4.1 impairs perineural K^+^ homeostasis, potentially contributing to motor neuron hyperexcitability in the *SOD1*^*G93A*^ transgenic mouse model [123, 124]. Dysfunction of astrocytic sodium–potassium-ATPase (NKA) has also been reported in neurodegenerative disease model [125]. Astrocytes also play an important role in maintaining Ca^2+^ homeostasis in the synaptic cleft [126]. Astrocytes express the sodium-calcium exchanger NCX to export Ca^2+^ to the synaptic cleft, at the expense of the Na^+^ gradient [127]. However, during neural hyperactivity, the activity-dependent Na^+^ transients in astrocytes switch NCX to a “reverse mode” by mediating Ca^2+^ import and Na^+^ export, dictating Ca^2+^ homeostasis in the synaptic cleft [127].

Thirdly, dysfunctional astrocytes lose their ability to provide metabolic support for neurons (Fig. 3C). Astrocyte-neuron metabolic coupling provides fundamental energy support for neuronal activity. Disrupting the expression of the astrocytic lactate transporters monocarboxylate transporter 4 (MCT4) or MCT1 has been reported in the *SOD1*^*G93A*^ mouse model, which is accompanied by a decrease in lactate level [128]. Primary astrocyte-motor neuron co-cultures from *SOD1*^*G93A*^ mice further confirmed that the significant metabolic dysfunction of astrocytes has been linked with motor neuron excitotoxicity [129].

Moreover, activated astrocytes release less neurotrophic factors like BDNF and GDNF. Instead, they produce abnormal levels of cytokines and proinflammatory substances, including TGF-β1, IFN- α, IFN- γ, IL- 6, ROS, NO, which can cause oxidative stress and inflammation, further contributing to motor neuron hyperexcitability (Fig. 3D) [130–133].

In conclusion, abnormally activated astrocytes lose their supportive functions and gain a more toxic function in ALS, which may contribute to the cortical hyperexcitability. The activated astrocytes become less effective in maintaining neurotransmitter and ion balance in the synaptic cleft or providing metabolic support, and in turn, release neurotoxic factors. Further research into the mechanisms of astrocyte-mediated neuronal activity change is likely to reveal new insights into the role of astrocytes in neurodegenerative diseases.

#### Oligodendrocytes

Oligodendrocytes are a specialized glial cell in the CNS that create myelin sheaths around axons [134, 135]. The myelin sheath is essential for action potential conduction and synchrony coordination, as it allows for rapid, efficient, and fine-tuned conduction (saltatory conduction) of action potentials along the axon. The myelin sheath also protects axons from damage, prevents the loss of signaling between neurons, and provides metabolic and neurotrophic support to axons [135]. Gaps in the myelin sheath, called nodes of Ranvier, have high concentrations of Na^+^ and K^+^ ion channels [136]. This allows for the rapid flow of ions into the axon and efficient action potential propagation.

In the last few years, accumulating evidence has indicated that oligodendrocyte dysfunction and myelin damage play an important role in neurodegenerative diseases, including ALS [137–141]. The role of oligodendrocytes in ALS have been comprehensively reviewed recently [142, 143]. Here, we mainly highlight the evidence regarding how dysfunctional oligodendrocytes contribute to motor neuron hyperexcitability (Fig. 4).

Massive myelin disorganization has been observed by electron microscopic (EM) examinations at the presymptomatic stage in *SOD1*^*G93A*^ transgenic mouse model, [144]. The damaged myelin contains decreased lipids, phospholipids, cholesterol and cerebrosides [144], suggest a loss of support function. This presymptomatic myelin change has been further confirmed by the global gene expression analysis of *SOD1*^*G93A*^ mice [145]. The defective oligodendrocytes and myelin may contribute to the UNM hyperexcitability through different mechanisms.

Firstly, defective oligodendrocytes lose their ability to monitor the health of the myelin sheath and make repairs when necessary (Fig. 4A). Myelination principally controls action potential speed along the axon and thus dysregulated myelin likely impairs electric conduction and neuronal activity. Myelin plasticity enables the fine-tuning of conduction velocity of action potentials, which is critical for establishing motor neuron synchronization within the motor cortical network [146–149]. Demyelination of layer V pyramidal neurons in the primary somatosensory regions resulted in loss of saltation and a broad presynaptic action potential in combination with reduced velocity [150]. Therefore, it is reasonable to speculate that changes in number, distribution, length, and thickness of myelin sheaths directly lead to disrupted action potential conduction along the axon and UMN excitability.

Secondly, defective oligodendrocytes become less effective in regulating the composition and structure of the nodes of Ranvier to ensure efficient nerve impulse conduction (Fig. 4B). Physiologically, myelin interacts with axonal proteins to form paranodal junctions, which is critical for node maintenance [151]. Mature nodes express NaV1.6 [152], and demyelination induced paranodal junction destabilization leads to a significant disruption of Na^+^ channel clustering in the node [151]. Accumulating evidence indicates axonal dysfunction in ALS [153]. However, the molecular and structural change of nodes of Ranvier in ALS is not clear. Future studies are needed to elucidate the pathology of nodes of Ranvier in ALS, as well as the potential role of myelin damage.

In addition, damaged oligodendrocytes become less effective in the regulation of the extracellular ion homeostasis. Oligodendrocytes play an important role in regulating white matter K^+^ buffering by clearing extracellular K^+^ [154]. A conditional knockout of the inwardly rectifying K^+^ channel Kir4.1 in mouse oligodendrocytes slows the clearance of extracellular K^+^, leading to neuronal hyperexcitability [154].

Thirdly, dysregulated oligodendrocytes impair metabolic support critical for maintaining axon integrity (Fig. 4C). Oligodendrocyte-derived metabolic support is mainly through providing lactate, which is transported into the periaxonal space by the oligodendrocytes MCT1 and then taken into the axon by neuronal MCT2 [155, 156]. MCT1 is highly enriched within oligodendroglia and a decrease of this transporter has been reported in patients with ALS and mouse models of ALS [155]. In addition, glutamate released from highly activated neurons can activate oligodendrocyte NMDA receptors, enhancing oligodendroglial glucose utilization and lactate supply for fast spiking axons [157]. Defective oligodendrocytes cannot meet the high energy demand of motor neuron, eventually leading to progressively axonal degeneration and changing neuronal excitability. Moreover, in ALS, damaged oligodendrocytes provide less neurotrophic support to axons due to decreased production of BDNF, GDNF, and insulin-like growth factor-1 (IGF-1) [158].

In conclusion, defective oligodendrocytes become less effective in maintaining the myelin sheath, providing metabolic and neurotrophic support, regulating the extracellular fluid environment, and maintaining the health of nodes of Ranvier. Changes in the function of oligodendrocytes may contribute to the development of neuronal hyperexcitability in ALS.

## Microglia play a potential role in regulating neuronal activity in ALS

Microglia are the principle immune cells that survey the surrounding microenvironments to maintain the homeostasis of the CNS. In the context of CNS disorders, microglia act as the first line of defense against damage by increasing their phagocytic ability and mediating repair [159–163]. Microglia activation is one of the hallmarks of ALS [164–167]. Activated microglia have been shown to have both detrimental and protective function in ALS [168, 169]. The general role of microglia in ALS has been discussed in previous reviews [170–173]. In this section, we will focus on the role of microglia in UMN hyperexcitability. Specifically, we will discuss the potential role of microglia in controlling neuronal hyperactivity, based on the growing body of evidence revealing that microglia are crucial in monitoring and regulating neuronal activity in both physiological and disease conditions (Fig. 5).

It is well known that activated microglia may contribute to motor neuron hyperexcitability by releasing proinflammatory and neurotoxic factors, including cytokines, reactive oxygen species, and proteases [174, 175]. These molecular signals can directly impact neuronal activity by altering the strength of synaptic connections [176, 177]. For example, SOD1^G93A^ microglia assaulted intact synapses, leading to axonal damage and motor neuron death through classical NF-κB pathway in ALS [178, 179]. Interestingly, microglia may also trigger the activation of other immune cells or astrocytes to indirectly affect neuronal activity [180–182]. Particularly, microglia can produce TNF-α, IL-1α and C1q to trigger neurotoxic reactive astrocytes in neurodegenerative diseases [183], which may act as an alternative mechanism to trigger neuronal hyperexcitability (Fig. 3D).

Although to what extent microglia directly affect UMN hyperactivity in ALS is unknown, the role of microglia in sensing and regulating neuronal activity has been emerging. Here we summarize the potential mechanisms based on the evidence derived from other disease models, which may provide new insights into the understanding of the function of microglia in neurodegenerative diseases. The general mechanisms regarding how microglia sense and regulate neuronal activity have been comprehensively reviewed previously [184]. Here we specifically discuss this topic in the context of ALS.

The first question is regarding how microglia sense UMN hyperexcitability. ATP and purinergic signaling is involved in the canonical pathway that mediate microglial response to neuronal hyperactivity in seizures and neuropathic pain conditions [185, 186]. P2Y12 is a purinergic receptor for adenosine diphosphate (ADP) that is exclusively expressed by microglia in the CNS. Hyperactivated motor neurons may release ATP into the extracellular space through vesicular and channel [e.g. volume-activated anion channels (VAAC), P2X7, Pannexin 1 channel (Panx1)] mediated release (Fig. 5A). Extracellular ATP will be hydrolyzed into ADP and then sensed by P2Y12 receptors, recruiting microglia processes towards this ATP source. Motor neuron and astrocyte-mediated ATP release has been shown to be increased in ALS [187–189]. How microglia purinergic signaling changes in ALS has not been fully understood. Other mechanisms may also be involved in microglia responses to neuronal hyperactivity, including membrane depolarization [190, 191], Ca^2+^ signaling [192], fractalkine signaling [193, 194], neuromodulators [195] and complement signals [196].

Once microglia processes are recruited into the close position of neuronal element, it can regulate neuronal activity through different mechanisms. Firstly, microglia can shape UMN hyperexcitability through synaptic pruning, a process by which microglia remove redundant synapses to regulate neural circuit function (Fig. 5B). Microglia-mediated synaptic pruning is critical during development to form well-tuned neural circuit, which is thought to be disrupted in neurological disorders like autism and schizophrenia. In addition, it has been recently shown that microglia promote synaptic pruning in AD [197]. In the context of ALS, it has been reported that C9orf72 deficiency transforms microglia from a homeostatic signature to an inflammatory state, leading to microglia-mediated synaptic loss [198]. The molecular components that mediate synaptic pruning include completement [197], CX3CR1 [199], and TREM2 [200, 201]. Several key questions remain to be addressed in this regard: 1) since weaker synapses are preferentially pruned by microglia, whether the neuronal hyperactivity can trigger synaptic pruning in ALS; 2) what would be the molecular signal released from neurons that initiates microglia interactions with the synapse; 3) in vivo imaging approaches should be applied to directly observe the synaptic pruning in ALS progression.

Secondly, microglia can establish physical contact with neurons and affect the functioning of key channels to fine-tune neuronal responses (Fig. 5C). For example, the processes of bipolar/rod-shaped microglia were aligned with the apical dendrites of pyramidal neurons in Huntington disease (HD) brain [174], indicating that microglia have the potential to regulate the neuronal activity through direct interaction. Previous studies have demonstrated the P2Y12-dependent close interaction between microglial processes and neuronal dendrites or cell bodies [185, 202]. In addition, detailed structural analysis showed the purinergic junction that microglia contact neuronal somatic membranes at Kv2.1/2.2 clustering [202]. The function of this specialized purinergic junction is proposed to be neuroprotective by reducing neuronal activity [185, 202]. However, how purinergic junctions dampen neuronal activity is still largely unknown. In future studies, it is important to determine how microglia establish direct interaction with different compartments of neurons and the function of these interaction patterns in ALS.

Thirdly, microglia may also regulate UMN excitability through contact with the axon (Fig. 5D) [203]. The axon initial segment (AIS), at which action potentials are initiated and modulated in neuron, is an important regulator of neuronal excitability. Interestingly, there is a distinct subset of microglia that specifically interact with AIS through development to adulthood in the cortex [204]. Of note, the AIS-microglia interactions are more specific to pyramidal neurons in cortical layer V [204], suggesting neuronal-subtype-specific functions. Activated microglia become less effective in forming functional association with AIS after brain injury [204], indicating the AIS-microglia interaction is a dynamic process dependent on brain conditions. Another interesting study showed that neuronal activity increases microglial interaction with nodes of Ranvier through potassium release to mediate axon repair [205]. Evoking repetitive action potentials in cortical pyramidal neurons recruits microglial processes to affected axons [206]. This interaction rescues neurons from excitotoxicity by preventing excess depolarization, suggesting a microglia-mediated neuroprotective action against neuronal hyperactivity.

Besides direct interaction with AIS and nodes of Ranvier, microglia seem to regulate chandelier cell (ChCs) axo-axonic synaptogenesis [207]. ChCs are a unique subset of cortical GABAergic interneuron. They form axo-axonic synapses exclusively on the AIS of pyramidal neurons, which provide precise inhibitory control to pyramidal neurons [208]. Thus, microglia-mediated regulation of ChCs might be crucial for precise control over neocortical pyramidal neurons firing. In the context of ALS, future studies are needed to determine the dynamic change of axon–microglia interactions in the disease progression, the functional significance of this interaction in UMN hyperexcitability, and the mechanisms mediating microglial contact with axon.

More recently, microglia have been reported to drive negative feedback to protect neurons from excessive activation in health and disease (Fig. 5E) [209, 210]. In particular, extracellular ATP converts into AMP by microglial ATP/ADP-hydrolyzing ectoenzyme CD39. AMP serves as a substrate for the microglial enzymes CD73 to generate adenosine (ADO), which suppresses neuronal activity by adenosine A1 receptors (A1Rs) [209]. Future studies need to test whether this mechanism also applies in the context of ALS.

Overall, microglia may exhibit both detrimental and protective functions in UMN hyperexcitability. The heterogeneous function of microglia highlights the complex interplay between microglia and neurons in the development and regulation of neuronal hyperexcitability in ALS. Further research is needed to fully understand the mechanisms by which microglia sense and regulate UMN hyperactivity, and to determine how this process may be harnessed to improve microglia-mediated neuroprotective effects. By better understanding the changes in microglial function in ALS, it may be possible to develop new therapeutic strategies targeting microglia to rescue the motor neuron hyperexcitability in ALS.

## Implications from single-cell omics evidence in ALS pathogenesis

The advent of single-cell mono or multi-omics technologies has sparked a technological revolution, offering novel insights into the molecular mechanisms underlying various physiological and pathological processes in different diseases, including ALS [211].

Mono-omics approaches, such as single-cell/single-nucleus transcriptomics and single-cell proteomics, have provided high-resolution views of single-cell heterogeneity and functional cell states in the motor system affected by ALS [212]. Specifically, single-cell transcriptional profiling of the primary motor cortex from ALS patients revealed significant transcriptional alterations in Betz cells and long-range projecting L3/L5 cells. Dysregulated pathways in excitatory neurons were found to be associated with stress response, ribosome function, oxidative phosphorylation, synaptic vesicle cycle, endoplasmic reticulum protein processing, and autophagy, which potentially contribute to motor cortical hyperexcitability in ALS [213]. Moreover, post-mortem tissue RNA-seq transcriptomes from the frontal cortex, temporal cortex, and cerebellum of FTLD-TDP patients confirmed the transcriptional profile changes of motor neurons along with alterations in other cell types, including microglia, astrocytes, oligodendrocytes, endothelial cells, and pericytes [214, 215]. Notably, cortical neuronal loss was strongly correlated with increased microglial and endothelial cell gene expression [214]. Similarly, single-cell RNA sequencing of human C9ORF72 ALS/FTD brain organoid slices revealed distinctive transcriptional, proteostasis, and DNA repair disturbances in astroglia and neurons [216]. Another study utilizing SOD1 iPSC (Induced Pluripotent Stem Cell)-derived motor neurons identified a disease-relevant transcriptional network change, highlighting the TGF-β signaling pathway as a key driver for motor neuron degeneration [217]. Additionally, transcriptomic analysis of the spinal cord in SOD1^G93A^ mice revealed dysregulation of lipid metabolic pathways in ALS [218].

While the utilization of single-cell proteomics in ALS remains limited, a recent study described single-cell proteomic profiling of postmortem human spinal motor neurons from ALS patients, uncovering differential protein abundance profiles related to oxidative phosphorylation, mRNA splicing and translation, and retromer-mediated vesicular transport when comparing motor neurons with or without obvious TDP-43 cytoplasmic inclusions [219].

Multi-omics approaches have emerged as powerful tools for comprehensively understanding ALS pathogenesis at different molecular levels, encompassineng gomics, epigenomics, transcriptomics, proteomics, and metabolomics [211]. In a recent study, single nucleus multiome analysis was performed in the prefrontal cortex of C9orf72 ALS/FTD patients, revealing distinct molecular changes in various cell types during disease progression. Notable findings included alterations in myelin protein components in premature oligodendrocytes, glucose/glycogen metabolism in astrocytes, and microglial reactivity. Moreover, layer 2–3 cortical projection neurons with high expression of CUX2/LAMP5 were found to be more vulnerable, offering valuable insights into the mechanisms underlying ALS pathology [220].

These technological advancements in single-cell omics have significantly advanced our understanding of disease mechanisms, revealing potential therapeutic targets for future ALS treatments. Further exploration and validation of these findings hold promise for improving the prognosis and management of ALS patients.

## Conclusion

In conclusion, the mechanisms underlying UMN hyperexcitability in ALS, as well as their downstream consequences are still incompletely understood. Both cell-autonomous and non-autonomous effects are believed to contribute to the UMN hyperexcitability, which holds great promise for novel treatment targets. Changes in the intrinsic properties of motor neurons as well as the function of glial cells play a crucial role in the development of motor neuron hyperexcitability of ALS. Of note, the role of microglia in regulating neuronal activity is increasingly being recognized. Understanding the changes in glial cell function in ALS may lead to a better understanding of the mechanisms underlying the cortical hyperexcitability. Studying glia-neuron interaction in ALS may promote the development of new treatments that target glial cells at early stages to slow the progression of the disease.

## Data Availability

The datasets during and/or analysed during the current study available from the corresponding author on reasonable request.
